# Gegen Qinlian Decoction treatment of asymptomatic hyperuricemia by targeting circadian immune function

**DOI:** 10.1186/s13020-023-00775-z

**Published:** 2023-06-27

**Authors:** Xiaojun Wang, Xuanqi Liu, Qiushuang Gao, Xuchao Gu, Guannan Zhang, Zhiyuan Sheng, Tao Wu, Zheling Su, Wenhao Wang, Maoqing Ye

**Affiliations:** 1 Department of Traditional Chinese Medicine, Huadong Hospital Affiliated to Fudan University, No 221West Yan-An Road, Shanghai, 200040 China; 2 Shanghai Key Laboratory of Clinical Geriatric Medicine, Huadong Hospital Affiliated to Fudan University, No 221 West Yan-An Road, Shanghai, 200040 China; 3 Department of Respiratory and Critical Medicine, Huadong Hospital Affiliated to Fudan University, Shanghai, 200040 China; 4 China Pharmaceutical University, Nanjing, 210009 China; 5 Department of Immunology, Key Laboratory of Immune Microenvironment and Disease, Nanjing Medical University, Nanjing, 211166 Jiangsu China; 6 Department of Urology, Huadong Hospital Affiliated to Fudan University, Shanghai, 200040 China; 7 Department of Nephrology, Yueyang Hospital of Integrated Traditional Chinese and Western Medicine, Shanghai University of Traditional Chinese Medicine, Shanghai, 200437 China; 8 Department of Cardiology, Huadong Hospital Affiliated to Fudan University, Shanghai, 200040 China

**Keywords:** Gegen Qinlian Decoction, Hyperuricemia, Circadian clock gene, Innate lymphoid cells

## Abstract

**Background:**

The Gegen Qinlian Decoction (GGQLD) is a renowned traditional Chinese medicinal formula that has been used for centuries to effectively treat asymptomatic Hyperuricemia (HUA). This study aims to investigate the underlying mechanism of GGQLD's therapeutic effects on HUA.

**Methods:**

The study enrolled a total of 25 healthy participants and 32 middle-aged and elderly individuals with asymptomatic HUA. All asymptomatic HUA participants were treated with GGQLD. Venous blood samples were collected from all participants to isolate peripheral blood mononuclear cells (PBMCs), which were then analyzed for biological profiles using flow cytometry. Network pharmacology analysis was utilized to identify the potential pathways involved in the therapeutic effects of GGQLD. Transcriptomic patterns of cultured proximal tubule epithelial cells (PTECs) were evaluated via bulk RNA-seq, and critical differentially expressed genes (DEGs) were identified and verified through ELISA. Molecular docking and molecular dynamics (MD) simulation were employed to investigate the potential compounds in GGQLD that may be involved in treating HUA.

**Results:**

Network pharmacology analysis revealed that immune-related pathways might be involved in the therapeutic mechanism of GGQLD. RNA-seq analysis confirmed the involvement of innate lymphoid cell (ILC) development-related genes and clock genes. Polychromatic flow cytometric analysis demonstrated that GGQLD treatment reduced the proportion of ILC3s in total ILCs in asymptomatic HUA patients. ELISA results showed that GGQLD treatment reduced the levels of activating factors, such as ILC3-IL-18 and IL-1β, in the plasma of HUA patients. GGQLD was also found to regulate circadian clock gene expression in PBMCs to treat asymptomatic HUA. Furthermore, the interaction between 40 compounds in GGQLD and HDAC3 (Histone Deacetylase 3), NLRP3 (NOD-like receptor protein 3), RORA (RAR-related orphan receptor A), and REV-ERBα (nuclear receptor subfamily 1) revealed that GGQLD may regulate ILCs and clock genes to treat asymptomatic HUA.

**Conclusions:**

The regulation of circadian clock gene expression and the proportion of ILC cells may be involved in the therapeutic effects of GGQLD on asymptomatic HUA patients.

**Supplementary Information:**

The online version contains supplementary material available at 10.1186/s13020-023-00775-z.

## Introduction

Hyperuricemia (HUA) is a metabolic disorder characterized by a clinical state in which the concentration of urate in the blood exceeds the solubility threshold (≥ 420 µmol/L in males, ≥ 360 µmol/L in females). HUA is often associated with other medical conditions, such as coronary heart disease, hypertension, diabetes, and hyperlipidemia. Additionally, the incidence of HUA is known to be closely related to age, gender, and body weight [1]. Asymptomatic HUA is characterized by hyperuricemia without gout attacks. Due to its non-specific clinical symptoms, it has not received consistent research attention in recent decades [2]. In recent years, the incidence rate of HUA in China has rapidly increased due to the fast-paced lifestyle and work demands [3]. Numerous epidemiological and clinical studies have shown that HUA is an independent risk factor for predicting the development of metabolic syndrome, kidney disease, and cardiovascular disease [4, 5]. In specific types of epithelial cells, soluble urate can induce oxidative stress and activate inflammatory signaling pathways through specific mechanisms. Recent studies have reported that exposure to high levels of soluble urate can alter the epigenetics of innate immune cells [6], resulting in the secretion of pro-inflammatory factors and the enhancement of continuous inflammation and hyperresponsiveness.

Overall, traditional chinese medicine treatments have the benefit of providing long-lasting and stable therapeutic effects for various metabolic diseases and immune-mediated inflammatory disorders [7]. Gegen Qinlian Decoction (GGQLD) is a classic ancient medicinal prescription that was initially documented in the Treatise on Febrile Diseases by Zhang Zhongjing over 1800 years ago, towards the end of the Eastern Han Dynasty [8]. The GGQLD prescription includes *Pueraria montana* var. lobata, *Scutellaria baicalensis*, *Coptis chinensis,* and *Glycyrrhiza uralensis*. While numerous experimental and numerical studies have already confirmed the significant role of GGQLD in immune protection, anti-inflammatory response, and improving metabolism in metabolic and inflammatory diseases, its relative molecular mechanism remains unclear [9–11].

Recent studies have highlighted the crucial involvement of circadian clock genes in metabolic diseases. Furthermore, evidence suggests that the occurrence of HUA is closely associated with circadian rhythm disorders. However, the precise role of circadian rhythm in HUA is not yet fully understood [12]. Recent research has shown that the circadian clock regulates the expression and activation of NOD-, LRR-, and pyrin domain-containing protein 3 (NLRP3), which in turn controls the secretion of IL-1β and IL-18 in various tissues and immune cells, particularly macrophages [13]. Our previous study confirmed that GGQLD can improve the inflammatory state of HUA patients by reducing IL-1β through inhibiting NLRP3. Therefore, this study aims to investigate whether GGQLD can regulate the circadian clock genes to inhibit NLRP3 in HUA.

The innate lymphoid cell (ILC) family consists of ILC1, ILC2, and ILC3, which are derived from ILC progenitors (ILCP) through various transcription factors. Recent research has shown that ILCs can interact with epithelial cells to maintain epithelial homeostasis and play a protective role in the organism [14]. When subjected to chronic stimuli such as high levels of uric acid (UA), innate lymphoid cells (ILCs) can become activated, which exacerbates the development of inflammatory diseases. While some researchers have demonstrated that natural killer (NK) cells are abnormally increased in patients with HUA, the role of ILCs in the pathogenesis of HUA has not been extensively studied [15]. Previous studies have shown that Huangqin decoction, another traditional Chinese medicine, can promote the regeneration of ILC3 in patients with enteritis [16]. However, it is still unclear whether GGQLD has a similar function.

Numerous studies have now confirmed the crucial role of GGQLD treatment in metabolic syndrome [17, 18]. Our research discovers the effect of GGQLD on immune cells for improving the metabolic disorder to treat HUA. The results suggest that GGQLD may regulate ILCs and some circadian clock family genes in PBMC of asymptomatic HUA patients. To further investigate the specific effects of GGQLD on these targets, molecular docking technology was used to explore the interaction of 40 compounds in GGQLD with circadian clock gene REV-ERBα, HDAC3, NLRP3, and RORA (RAR-related orphan receptor A), respectively. These results provide a new theoretical foundation for elucidating the circadian rhythm of immune cells regulated by GGQLD in the treatment of HUA.

## Materials and methods

### Enrollment of participants

The current study is registered in the Chinese Clinical Register (ChiCTR2000038257). From January 1st, 2020 to February 31st, 2021, a total of 25 healthy controls and 32 patients with asymptomatic HUA were enrolled at Huadong Hospital affiliated with Fudan University in Shanghai, China. Inclusion criteria were as follows: aged 50–75 and diagnosed with HUA (SUA > 420 µmol/L). Patients receiving anti-HUA treatment were required to wait two weeks for clearance, and only those with SUA > 420 µmol/L after clearance were included. Exclusion criteria for patients included: allergic physique or previous history of allergy to Benzbromarone or TCM, two-fold elevation of ALT in comparison with the normal upper limit, severe renal insufficiency, serious stiffness or deformity due to gouty arthropathy, clinically significant arrhythmia, and alcohol abuse history. Additionally, those with serious concurrent diseases in the hematopoietic system, liver, cerebrovascular system, or kidney, mental diseases, malignant cancers, those taking salicylate or aspirin (> 325 mg/d)-containing medications, hypouricemic medications, 6-mercaptopurine, or azathioprine, or those involved in additional clinical trials in the last three months were excluded. All asymptomatic HUA patients included in the study received GGQLD treatment (twice daily for four weeks), and SUA was used to assess the treatment's effectiveness. Approval for the protocol was obtained from the local Ethics Committee of Huadong Hospital affiliated with Fudan University (No. 20190037), and informed written consent was obtained from all participants.

### Preparation of GGQLD and High-Performance Liquid Chromatography (HPLC)

GGQLD was prepared using the boiling water extraction method on four dry herbal medicines: Gegen, Huangqin, Huanglian, and Zhigancao. The herbs were mixed in a ratio of 8:3:3:2 and soaked with distilled water (eightfold volume v/w) for 30 min. The mixture was extracted using a decoction pot twice (1 h and 40 min each time), and the supernatants were combined and concentrated to 1 g crude medicine/mL using a rotary evaporator. The crude extract was then preserved at 4 °C for in vitro experiments. Stigmasterol the bioactive ingredients with purities greater than 98%, were provided by Shanghai Yuanye Biotechnology Co., Ltd. (Shanghai, China). The chemical structure of stigmasterol is presented in Fig. 5A. The HPLC analysis confirmed the puerarin, baicalin, liquiritin, and berberine content in accordance with a previous study [17]. HPLC analysis was conducted using an Agilent 1200 HPLC1200 (Thermo Fisher Scientific, New York, United States). The detection wavelength for stigmasterol was set at 210 nm. The decoction was administered orally as a water extract.

### Soluble UA preparation

According to previous research, UA was dissolved in 1 M NaOH to achieve a final concentration of 50 mg/mL [19]. Subsequently, the solution was examined to confirm its lack of mycoplasma contamination. The solution was also filtered through a 22 µm pore size filter prior to use. Additionally, no visible crystals were observed under polarizing microscopy or during cell incubation.

### CCK8 Assay on the Viability of Cells Treated with Different Doses of GGQLD

The viability of PTECs treated with GGQLD at different doses was evaluated using CCK-8 assays (Beijing Solarbio Science & Technology Co., Ltd, China). PTECs were seeded at a density of 1 × 10^4^ cells/well in a 96-well plate and incubated for 1–2 days. The absorbance (OD) at 450 nm was measured using a microplate reader. All experiments were performed in duplicate. The Cell Counting Kit-8 (CCK8) was provided by Shanghai Jiwei Biological Technology (Shanghai, China).

### PTECs culture

The cell culture medium and human primary PTECs were obtained from ScienCell (San Diego, CA, USA), while UA was sourced from Sigma (St. Louis, MO, USA). The Epithelial Cell Medium, consisting of basal medium (500 ml), fetal bovine serum (10 ml, FBS), penicillin/streptomycin (5 ml), and epithelial cell growth supplement (5 ml), was used to culture human primary PTECs. The PTECs were incubated at 37 °C and 5% CO_2_ and subjected to a 24-h "growth arrest" period in serum-free medium before each experiment.

### RNA sequencing (RNA-Seq)

The PTECs were harvested before and after GGQLD treatment and washed twice with ice-cold PBS. Six samples were collected, three from the GGQLD group and three from the control group. Total RNA was extracted using TRIzol reagent (Takara, Dalian, China) following the manufacturer's instructions. mRNA libraries were constructed and sequenced by Nuohe Zhiyuan Technology Company (Beijing, China). Differential expression analysis of the data was performed using DESeq (version 1.30.0. False discovery rate (FDR) was used to adjust p-value with the application of significance thresholds of fold change (FC) ≥ 2 and P < 0.05.

### Bioinformatics analysis

Targets related to diseases were identified using DisGeNET (http://www.disgenet.org) and NCBI. The KEGG signaling pathway enrichment analysis of significant targets of GGQLD and HUA was conducted using the DAVID 6.8 database [20]. The protein–protein interaction (PPI) network was constructed using the STRING website, and hub genes were identified using STRING and Cytoscape software. Statistical significance was set at P < 0.05.

### The collection of human peripheral blood mononuclear cells

A total of 6 mL of venous blood samples were collected in sterile tubes containing ethylenediamine tetraacetic acid (EDTA) and processed immediately. Peripheral blood mononuclear cells were isolated using Ficoll-Paque solution (STEMCELL, British Columbia, Canada) via density-gradient centrifugation. The blood sample was layered carefully, and the interface layer containing PBMCs was obtained and washed twice with PBS. The cell pellets were then resuspended in PBS and incubated with a fluorescent antibody (FA).

### Flow cytometry assay

The biological analysis was conducted on fresh peripheral blood mononuclear cells (PBMCs) derived from blood samples. To assess the subsets of ILC1, ILC2, and ILC3 in peripheral blood among patients with elevated uric acid (UA), a flow cytometry assay was carried out. Briefly, 1 × 10^6^ cells were suspended in 100 µL phosphate-buffered saline (PBS) prior to antibody incubation. The cells were then incubated with Zombie Aqua fixable viability dye (BioLegend, San Diego, CA, USA), anti-human-CD45-APC/Fire 750 (BioLegend), anti-human-c-Kit-PE/Cy7, anti-human-CRTH2-PerCP/Cy5.5, anti-human-Lineage cocktail (CD3/14/16/19/20/56)-FITC, and anti-human-CD127-BV421 (all from BioLegend) at room temperature for 30 min in the absence of light. Following a wash with 2 mL PBS, the cells were resuspended in 500 µL PBS and examined using flow cytometry (FACS Aria™ II, BD Bioscience, NJ, China). List mode data files were analyzed using FlowJo™ 10 software. Total lymphocytes were identified based on forward and side scatter properties. The innate lymphoid cell (ILC) gate was defined by CD45 + Lineage-CD127 + expression. ILC subtypes were further distinguished as ILC1s (c-Kit-CRTH2-), ILC3s (c-Kit-CRTH2 +), and ILC2s (c-Kit +).

### Reverse transcription-polymerase chain reaction (RT-PCR)

Total RNA from samples was isolated using TRIzol® reagent (Invitrogen; Thermo Fisher Scientific, Inc., USA). The PrimeScript RT Reagent kit (Takara Biotechnology) was employed for cDNA synthesis through reverse transcription. Subsequently, Power SYBR Green (Takara Biotechnology) and the RT-PCR detection system were utilized to amplify specific cDNA fragments with corresponding primers. Data were expressed as relative levels. The 2-ΔΔCq method was used to determine relative gene expression, using β-actin as an internal reference. Furthermore, the primer sequences employed in the RT-PCR experiments are provided in Additional file 1: Table S4.

### Enzyme-linked immuno sorbent assay (ELISA) of IL-18 and IL-1β protein in plasma

Plasma was obtained from normal control subjects, asymptomatic hyperuricemia (HUA) patients, and asymptomatic HUA patients treated with GGQLD. The collected plasma samples were stored at –80 °C for subsequent protein analyses. A commercial assay kit (eBioscience, San Diego, USA) was employed to measure the plasma levels of IL-1β and IL-18 proteins, following the manufacturer's guidelines.

### Statistical analysis

Categorical variables were represented as counts and percentages and analyzed using the chi-square test in univariate analysis, while continuous variables were expressed as mean ± standard deviation (SD) and examined via t-test. All tests were two-sided, and a P-value of less than 0.05 indicated statistical significance. Data analyses were conducted using R software (Version 3.6.3).

### Molecular docking

To gain insights into the potential binding modes between the 40 compounds in GGQLD and the 4 significant targets (REV-ERBα, HDAC3, NLRP3, and RORA), molecular docking was conducted using AutoDock Vina [21]. The three-dimensional (3D) structures of HDAC3, NLRP3, RORA, and REV-ERBα, each complexed with potent ligands, were obtained from the Protein Data Bank (PDB codes: 4A69, 7ALV, 4S15, and 3N00, respectively). Initially, AutoDockTools 1.5.6 was employed to prepare all input files. For molecular docking, only one protein chain was retained in each crystal complex. Water molecules and solvent molecules were removed, and polar hydrogens were added to the system. For HDAC3, as it contains Zn^2+^, the charge of the Zn^2+^ ion in the PDBQT file of HDAC3 was assigned as + 2.0. Grid box coordinates in the XYZ directions were generated, centered on the ligand in the protein crystal, with lengths of 20 Å assigned in each direction. The Lamarckian algorithm was utilized to identify the optimal binding mode of the ligand molecule, setting the exhaustiveness to 8. Additionally, the maximum number of output conformations was set to 10, with a maximum allowable energy range of 3 kcal/mol. Docking results were processed using PyMoL.

### Molecular dynamic simulation

To evaluate the binding stability of the identified ligands with REV-ERBα, HDAC3, NLRP3, and RORA, molecular dynamics simulations were performed using the Gromacs 2019 package (Gromacs-2019.1, GROMACS Development Team, 2019) [22]. The CGenFF software was utilized to produce the str files for the ligands in this study [23].

The molecular dynamic simulation utilized the CHARMm36 force field (charmm36-mar2019.ff) [24] and topology files for the protein and ligands were generated through GROMACS. The protein–ligand complexes were solvated in a dodecahedron box with TIP3P water molecules and a 10 Å margin. To neutralize the system charge, sodium and chloride ions with a concentration of 0.154 M were added. The LINCS algorithm was used to restrict bond lengths of covalent bonds, while the particle mesh Ewald (PME) method measured long-range electrostatic connections. The solvated system was energy minimized using the steepest descent algorithm with a 5000-step cutoff. Subsequently, the system was heated from 0 to 300 K for 100 ps under NVT, followed by a 100 ps NPT simulation at 1 atm, both of which were subjected to harmonic restraints on the complex. Each complex was subjected to a production run of molecular dynamic simulation for 100 ns with a time-step of 2 fs. The GROMACS built-in tools were used to calculate the root-mean-square deviation (RMSD) values for each ligand and protein.

## Results

### The clinical characteristics of normal control and asymptomatic HUA patients

**Fig. 1:**
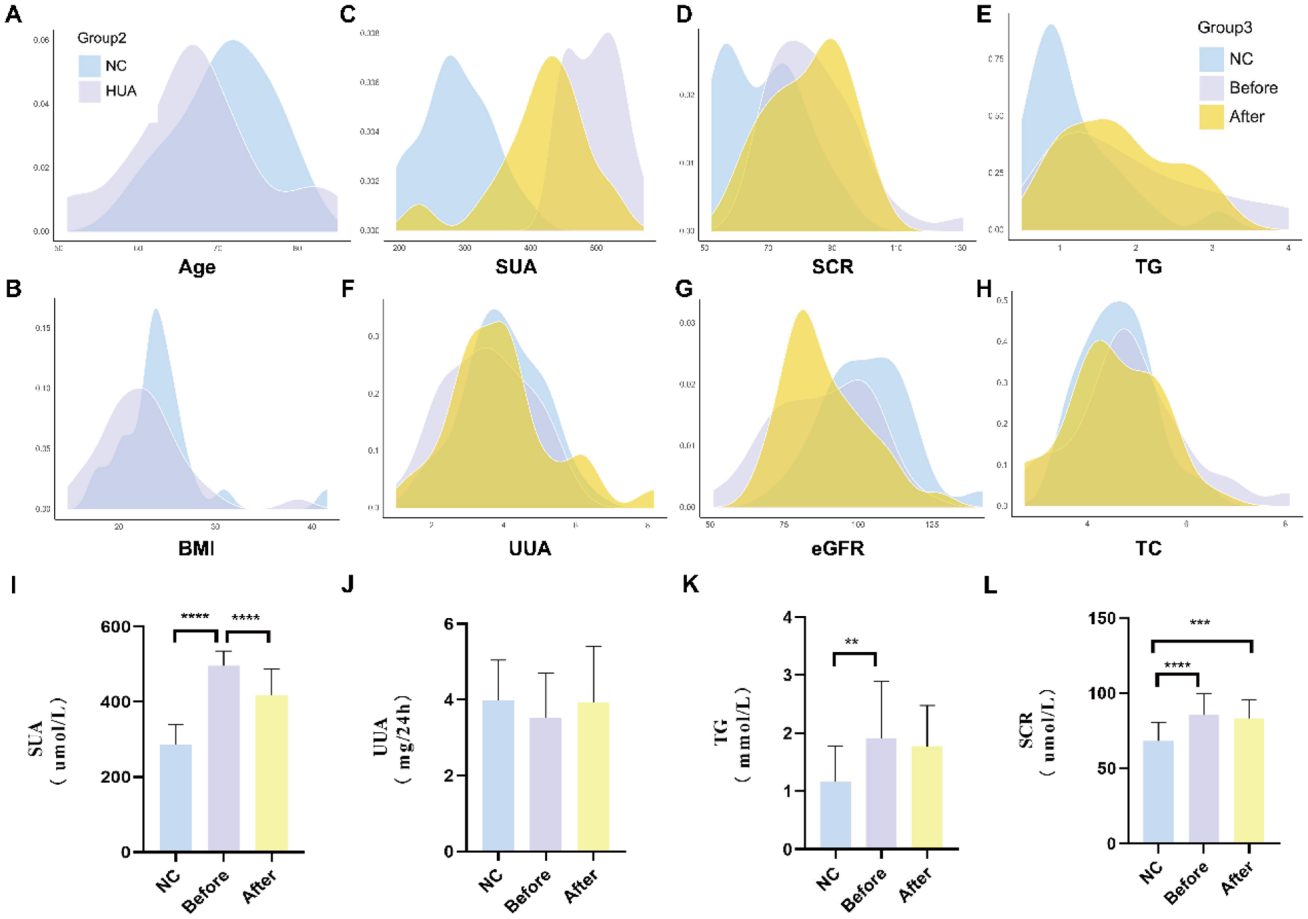
The difference of hemogram indices among normal control and HUA patients before and after GGQLD treatment. **A**, **B** Density plot shows the distribution of different baseline features concluding age and BMI between normal ones and asymptomatic HUA patients. **C**–**H** Density plot in three colors indicated the distribution difference of blood routine examination among normal control group(NC), HUA before GGQLD treatment(Before) and HUA after GGQLD treatment(After)—SUA, SCR, TG, UUA, eGFR, TC. **I**–**L** The bar chart indicated the comparison of SUA (*P* < 0.0001), UUA, TG and SCR. In all statistical plots, data are expressed as the mean ± SD, *p < 0.05, **p < 0.01, ***p < 0.001, ****p < 0.0001

Additional file 1: Table S1 presents the clinical phenotypes, including baseline indicators, hemogram indices, and uric indexes, of the enrolled population. Both healthy individuals and those diagnosed with asymptomatic HUA were included in the study, and dynamic changes before and after GGQLD treatment were observed. Figure 1 provides a visual representation of the change in clinical parameters among normal controls, asymptomatic HUA patients before and after GGQLD treatment. There were no significant statistical differences in age, BMI, TC, ALT, and AST between the normal controls and HUA patients. The mean age of controls was 70.880 ± 6.037, and that of the cases was 67.250 ± 8.332 years (*P* = 0.1461). The mean BMI of controls was 24.229 ± 4.639 (kg/m2), and that of the cases was 22.473 ± 4.610 (kg/m2) (P = 0.2844). The results indicated that GGQLD reduced the SUA level (Additional file 1: Table S1) from 496.100 ± 39.430 (µmol/L) to 417.900 ± 69.620 (µmol/L) (P < 0.0001) (Fig. 1I) and increased the UUA level (Fig. 1J) from 3.527 ± 1.183 (mmol/L) to 3.934 ± 1.468 (mmol/L) (Additional file 1: Table S1) of asymptomatic HUA patients in our study (n = 32). Additionally, the results showed that GGQLD could improve renal function, as evidenced by the decrease of SCR from 85.650 ± 14.070 (µmol/L) to 83.150 ± 12.530 (µmol/L) and the increase of eGFR (Fig. 1L) from 86.99 ± 16.51 (mL/min) to 88.55 ± 14.46 (mL/min). The hemogram index TG was also reduced (Fig. 1K) from 1.911 ± 0.981 (mmol/L) to 1.771 ± 0.706 (mmol/L), indicating that GGQLD might improve the metabolic status of asymptomatic HUA patients. Based on the clinical data, the potential value of GGQLD in reducing SUA levels in asymptomatic HUA patients will be discussed.

### Bioinformatics analysis and RNA-seq analysis of PTEC suggested that GGQLD may participate in the treatment of HUA through immune regulation

**Fig. 2:**
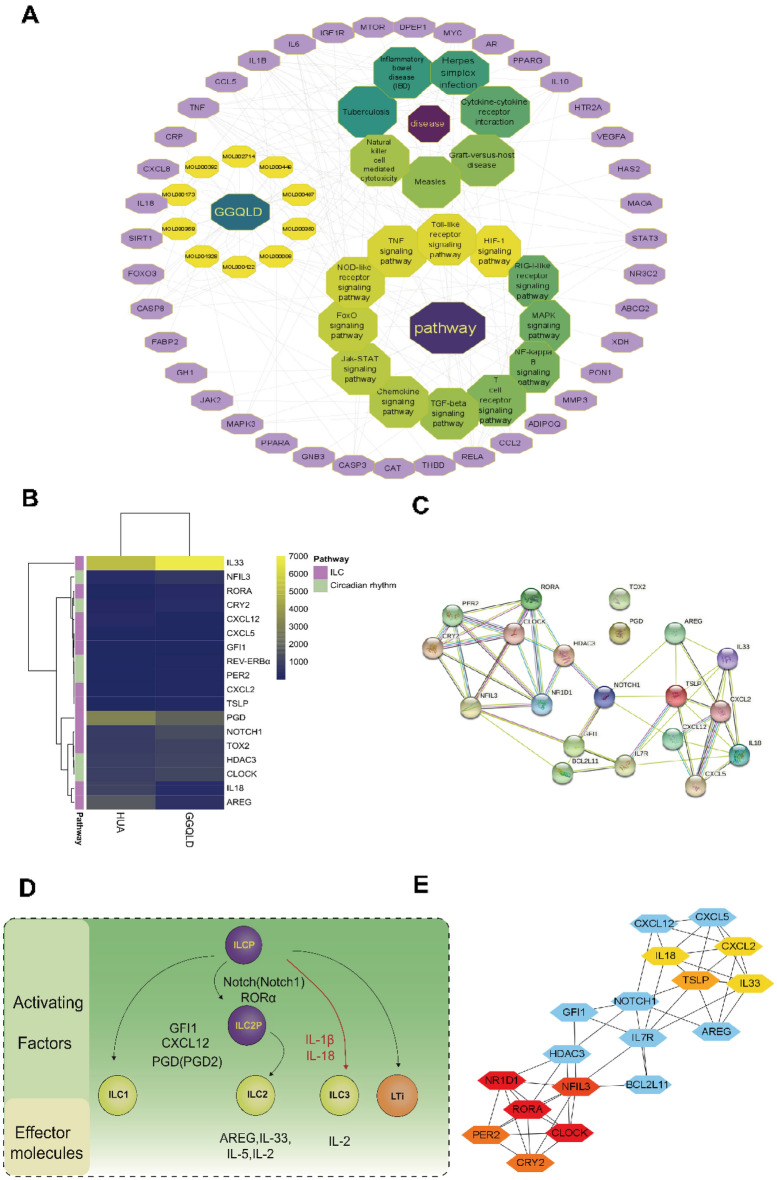
Bioinformation and RNA-seq analysis in PTECs suggested that the treatment of HUA with GGQLD may be related to innate lymphoid cells and clock genes. **A** The figure of network pharmacology analysis revealed the potential pathway of GGQLD in the treatment of HUA. **B** The RNA-seq analysis in PTECs stimulated with high uric acid before and after intervention by GGQLD shows the different expression in ILC development related genes and clock genes. **C** The diagram shows the key genes and cytokines involved in the biological process of ILC development. **D** *Protein–protein interaction and gene* co-expression network about the genes related to ILC development and circadian rhythm which obtained from the RNA-seq results. **E** 10 hub genes were identified based on the protein–protein network in **D** constructed in the STRING database with Cytoscape software

To identify the underlying targets and downstream signaling pathways of GGQLD in HUA treatment, we constructed an active component-target-disease-drug interaction network using Cytoscape 3.8.0. The network revealed that the common pathways involved were primarily related to the ‘T cell receptor signaling pathway’, ‘Toll-like receptor signaling pathway’, ‘HIF-1 signaling pathway’, ‘RIG-I-like receptor signaling pathway’, ‘MAPK signaling pathway’, ‘NF-kappaB signaling pathway’, ‘TGF-beta signaling pathway’, ‘Chemokine signaling pathway’, ‘JAK-STAT signaling pathway’, ‘FoxO signaling pathway’, ‘NOD-like receptor’ and ‘TNF signaling pathway’. The common diseases analyzed were inflammatory bowel disease (IBD), Herpes simplex infection, tuberculosis (TB), measles, and graft-versus-host disease (GVHD) (Fig. 2A). The immune-related pathways involved in the course of HUA by GGQLD treatment included the TGF-β signaling pathway, NOD-like receptor signaling pathway, T cell receptor signaling pathway, chemokine signaling pathway, TNF-signaling pathway, as well as Toll-like receptor signaling pathway, suggesting that GGQLD may improve immune defense function when challenged with external factors such as high concentrations of uric acid (Fig. 2A).

To further analyze the transcriptomic profiles based on bulk RNA-seq results obtained from a previous study [17], we compared differentially expressed genes (DEGs) screened from PTECs before and after GGQLD intervention. The results indicated that circadian rhythm family genes and ILC development-related genes were significantly altered (Fig. 2B). Critical genes and cytokines related to the development of ILCs are explained in Fig. 2D. Based on the DEGs from the bulk RNA-seq results in PTECs, a target protein–protein interaction (PPI) network was constructed using the STRING platform (Fig. 2C). Furthermore, a plugin of the Cytoscape software was used to select ten hub genes (RORA, NR1D1 (REV-ERBα), NFIL3, PER2, CRY2, IL-18, IL33, TSLP, and CXCL2) (Fig. 2E). IL-18 was identified as the activating factor of ILC3, while IL33 was the effector molecule of ILC2. RORA, TSLP, and NFIL3 were also related to the development of ILC2. NR1D1, REV-ERBα, PER2, and CRY2 were identified as circadian clock genes, as illustrated in Fig. 2D.

### GGQLD treatment regulates the ILCs proportion in PBMCs of HUA patients

**Fig. 3:**
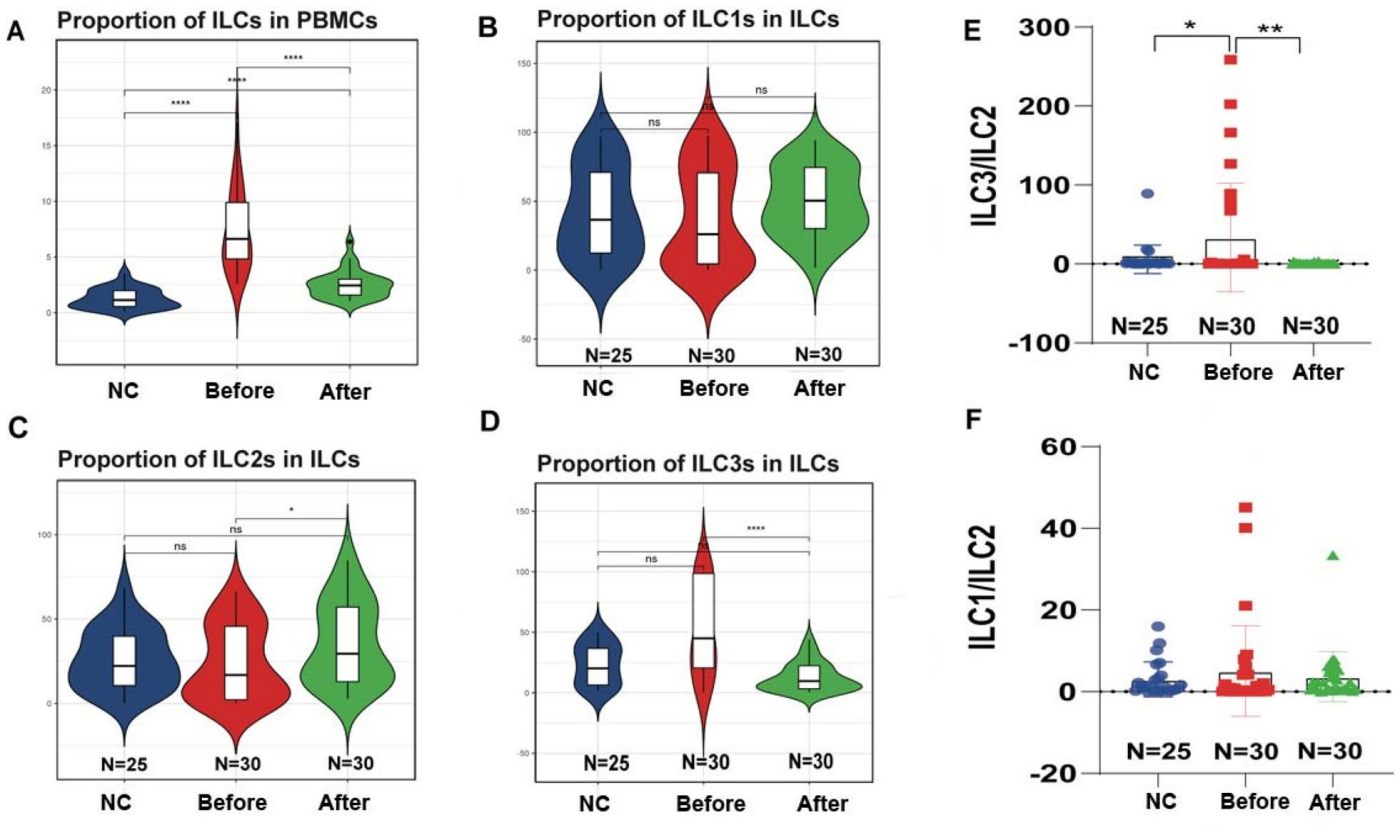
The difference of ILC number and the proportion among the normal control and HUA patients before and after GGQLD treatment. **A** The violin plot shows the proportion of ILCs in PBMCs. **B**–**D** The proportion of ILC1, ILC2 and ILC3 in total ILCs was displayed among NC, HUA before and after GGQLD treatment groups. **E**, **F** The two indices (ILC3-ILC2 ratio and ILC1-ILC2 ratio) were compared among NC, HUA before and after GGQLD treatment groups. In all statistical plots, data are expressed as the mean ± SD, *p < 0.05, **p < 0.01, ***p < 0.001, ****p < 0.0001

To determine the role of ILCs in asymptomatic HUA patients, PBMCs were collected from normal controls, asymptomatic HUA patients before and after GGQLD treatment, and compared in Table 1. The gating strategy for ILCs is shown in Additional file 1: Fig. S3 through flow cytometry. As expected, the total number of ILCs was elevated in asymptomatic HUA patients and reduced after GGQLD treatment. Specifically, the proportion of ILC subsets in PBMCs of asymptomatic HUA patients significantly increased (P < 0.0001) (Fig. 3A). There was no significant difference in ILC1 between the three datasets (Fig. 3B). However, the proportion of ILC2 subsets in ILCs significantly increased (P < 0.05) in PBMCs from HUA patients after GGQLD treatment (Fig. 3C). Additionally, the high proportion of ILC3 subsets in ILCs from HUA patients was significantly decreased (P < 0.0001) after GGQLD treatment (Fig. 3D). It can be inferred that the balance of the proportion of ILC2 and ILC3 (the ratio index-ILC3/ILC2) in ILCs may be altered in asymptomatic HUA patients (P < 0.05), while GGQLD improved the imbalance of ILC2 and ILC3 in ILCs (P < 0.01) (Fig. 3E). Furthermore, no significant difference in ILC1-ILC2 ratios was observed before and after GGQLD treatment in HUA patients (Fig. 3F).

**Table 1:** The number of innate lymphoid cells (ILCs) among three groups

	Normal control (N = 25)	HUA before GGQLD (N = 30)	HUA after GGQLD (N = 30)	*P*-value
ILC	1.265 ± 0.885	7.243 ± 3.594	2.523 ± 1.245	< 0.0001*
ILC1	41.972 ± 32.606	37.943 ± 36.289	49.895 ± 25.103	0.3361
ILC2	26.226 ± 17.918	23.957 ± 22.081	35.900 ± 24.283	0.0877
ILC3	31.802 ± 24.460	38.100 ± 39.037	14.205 ± 12.618	0.0040*
ILC1/ILC2	132.668 ± 648.069	5.078 ± 11.081	3.698 ± 6.146	0.3122
ILC3/ILC2	5.806 ± 17.946	33.584 ± 68.674	0.743 ± 0.991	0.0072*

^*^P ≤ 0.05

### GGQLD treatment regulates circadian clock genes expression in PBMCs of HUA patients

**Fig. 4:**
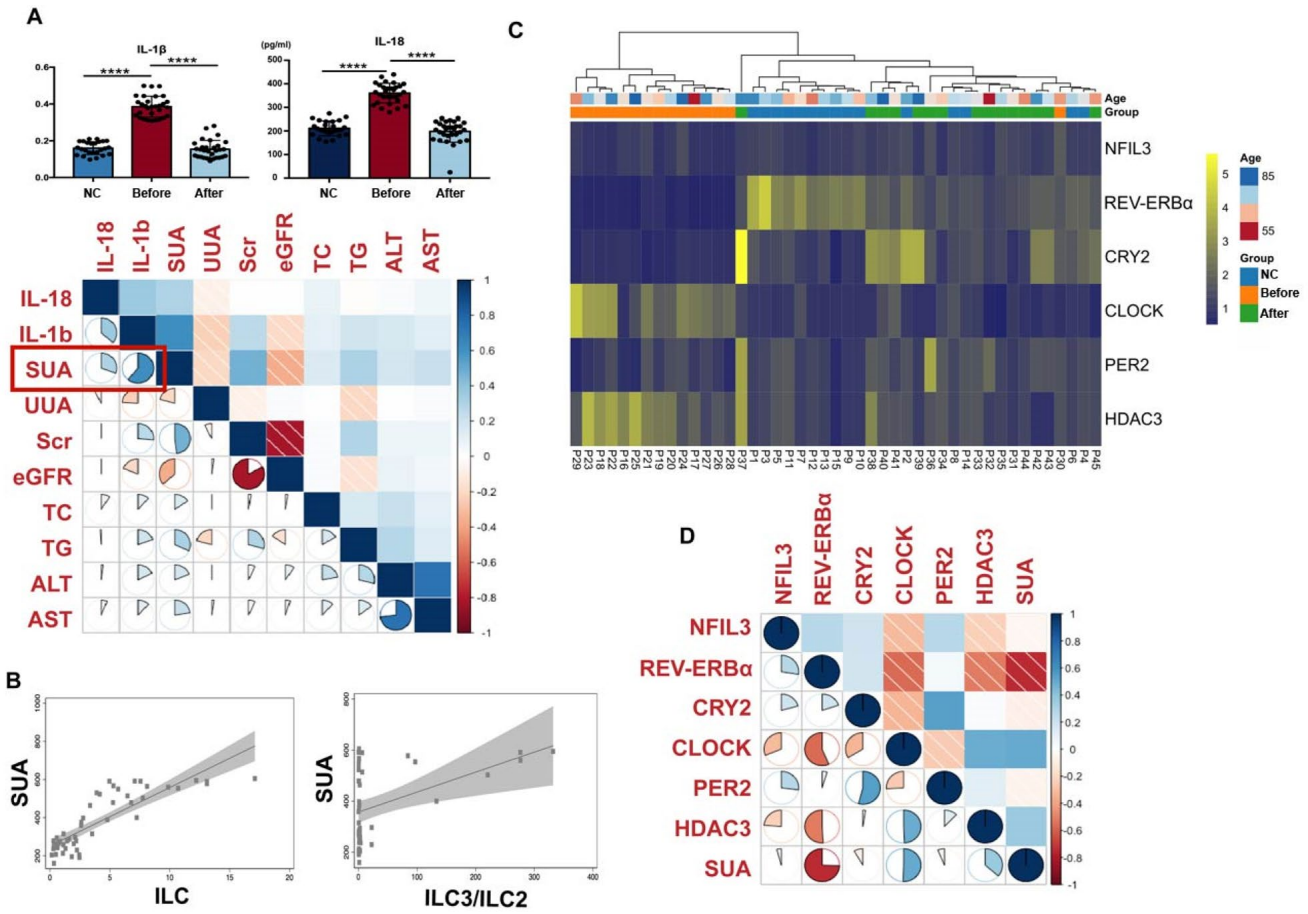
GGQLD may treat HUA by regulating clock genes in PBMCs. **A** Τhe bar plot shows the level of IL-18 and IL-1β in plasma from patients in NC, HUA before and after GGQLD treatment groups according to the results of ELISA. Regression coefficient matrix shows the relationship between cytokine (IL-18, IL-1β) and blood biochemical indexes. **B** Linear regression model shows the association between SUA levels and the proportion of ILCs, ILC3-ILC2 ratio in PBMCs. **C** Stratified by age and group (NC, HUA before and after GGQLD treatment), the heatmap shows the relative RNA expression of clock genes (NFIL3, REV-ERBα, CRY2, CLOCK, PER2 and HDAC3) in PBMCs. **D** Regression coefficient matrix shows the relationship between the level of biological clock genes and SUA levels. In all statistical plots, data are expressed as the mean ± SD, *p < 0.05, **p < 0.01, ***p < 0.001, ****p < 0.0001

The level of IL-18 and IL-1β in plasma from normal controls, asymptomatic HUA patients before and after GGQLD treatment were determined by ELISA (Fig. 4A). The activating factors of ILC3, such as IL-18 (P < 0.0001) and IL-1β (P < 0.0001), were significantly increased in asymptomatic HUA patients compared to normal control patients and decreased after GGQLD treatment. In the coefficient correlation matrix, IL18 and IL-1β showed the strongest association with the SUA level. According to Fig. 4B, the SUA level is associated with the proportion of ILCs (P < 0.001) and ILC3-ILC2 ratio (P = 0.0035) in the linear regression based on our results. Cluster analysis revealed that the circadian rhythm-related genes in asymptomatic HUA patients differed from those in normal individuals (Fig. 4C). Specifically, the relative RNA expression of CRY2 and REV-ERBα decreased, while the relative RNA expression of HDAC3 and CLOCK increased in PBMCs from asymptomatic HUA patients. There was no significant difference in PER2 and NFIL3 gene expression in PBMCs between normal control and HUA patients. Among the six circadian rhythm genes (HDAC3, CLOCK, PER2, CRY2, and NFIL3) explored, the most significant correlation was observed between the relative RNA expression of REV-ERBα and the SUA level. The relative RNA expression of REV-ERBα was negatively correlated with the SUA level, which was consistent with our prior RNA-seq results (Fig. 4D).

### Molecular docking study suggested the possible potential pharmacodynamic components for treating HUA in GGQLD

**Fig. 5:**
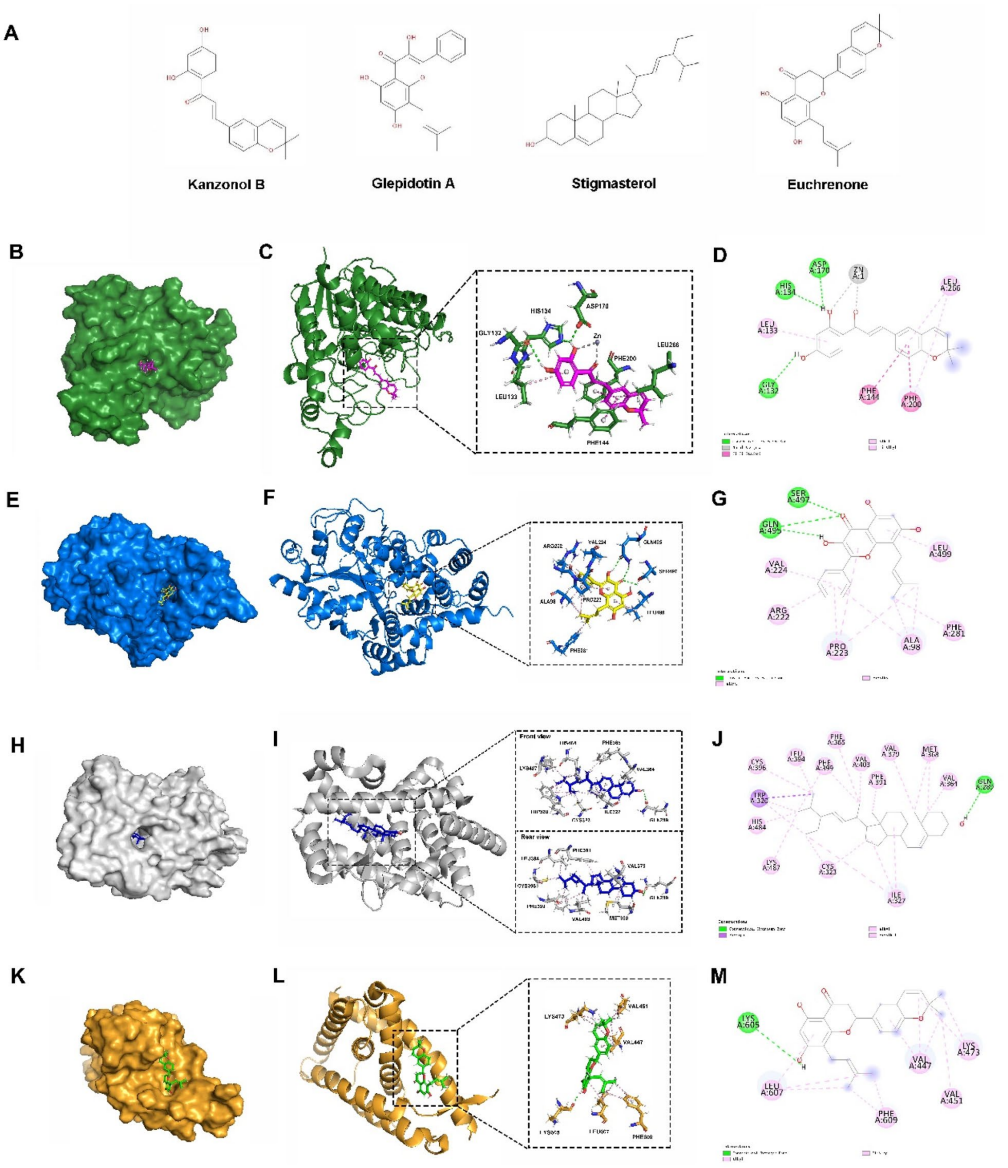
The docking results of selected compounds in GGQLD with HDAC3, NLRP3, RORA and REV-ERBα. **A** The structure of the compound Kanzonol B, Glepidotin A, Stigmasterol and Euchrenone. **B**, **E**, **H**, and **K** represent compound Kanzonol B, Glepidotin A, Stigmasterol and Euchrenone bound to active sites of HDAC3, NLRP3, RORA and REV-ERBα, respectively. **C**, **F**, **I**, and **L** represent the interactions between Kanzonol B, Glepidotin A, Stigmasterol and Euchrenone and HDAC3, NLRP3, RORA and REV-ERBα, respectively. **D**, **G**, **J**, and **M** represent2D ligand interactions diagram of Kanzonol B, Glepidotin A, Stigmasterol and Euchrenone bound to HDAC3, NLRP3, RORA and REV-ERBα, respectively. HDAC3, NLRP3, RORA and REV-ERBα are displayed as green, blue, light gray and light orange, respectively. Compound Kanzonol B, Glepidotin A, Stigmasterol and Euchrenone are shown as magenta, yellow, blue and green sticks. The π−π stacked interactions are displayed with magenta dashes. The π−alkyl and alkyl interactions are displayed with light pink dashes. The π−sigma interactions are displayed with purple dashes. The hydrogen bonds are shown as green dashes and zinc coordination are displayed with gray dashes

Tables 2, 3, 4, and 5 present the detailed interaction results of the top 5 compounds with high docking scores in GGQLD with HDAC3, NLRP3, RORA, and REV-ERBα, respectively. Notably, Kanzonol B had a high binding affinity for HDAC3 with a relatively high docking score of –8.8 kcal/mol. Figure 5B indicates that Kanzonol B binds to the active pocket of the HDAC3 protein. The association between ligands and Zn^2+^ ion is crucial for enhancing the inhibitory activity of the ligand against HDAC3 [25]. Compound Kanzonol B formed bidentate chelation with Zn^2+^ and engaged in hydrogen bond interactions with GLY132, HIS134, and ASP170, as well as hydrophobic interactions with LEU133, PHE144, FHE200, and LEU266 (Fig. 5C and D). Glepidotin A had a docking score of –8.9 kcal/mol against NLRP3 and was observed to bind to the NACHT domain of NLRP3 (Fig. 5E). Glepidotin A formed hydrogen bond interactions with SER497 and GLN495, and hydrophobic interactions with ALA98, ARG222, PRO223, VAL224, PHE281, and LEU499 (Fig. 5F and Fig. 5G). Compound Stigmasterol had a docking score of –11.6 kcal/mol against RORA and was found to bind to the ligand-binding domain of RORA (Fig. 5H). Stigmasterol formed hydrogen-bonding interaction with GLN289, and hydrophobic interactions with TRP320, CYS323, ILE327, VAL364, PHE365, MET368, VAL379, PHE391, LEU394, CYS396, PHE399, VAL403, HIS484, and LYS487 (Fig. 5I and J). For REV-ERBα, compound Euchrenone had a docking score of –8.1 kcal/mol and was observed to bind to the ligand-binding domain of REV-ERBα (Fig. 5K). Euchrenone formed a hydrogen bond interaction with LYS605 and hydrophobic interactions with VAL447, VAL451, LYS473, LEU607, and PHE609 (Fig. 5L and M).

**Table 2:** The docking results of top-ranked 5 compounds towards HDAC3

Compound	Docking score (kcal/mol)	H-Bond interactions	Hydrophobic interactions
Kanzonol B	− 8.8	GLY132, HIS134, ASP170	LEU133, PHE144, PHE200, LEU266
Rivularin	− 6.7	HIS134, HIS135, HIS172, TYR298	PHE144, CYS145, PHE200, LEU266
Liquiritin	− 6.5	HIS134, ASP170, HIS172	PHE144, LEU266
Calycosin	− 6.3	HIS134, ASP170	PHE144, CYS145, PHE200, LEU266, TYR298
Glypallichalcone	− 6.3	HIS172	LEU133, PHE144, CYS145, PHE200

**Table 3:** The docking results of top-ranked 5 compounds towards NLRP3

Compound	Docking score (kcal/mol)	H-Bond interactions	Hydrophobic interactions
Glepidotin A	− 8.9	GLN495, SER497	ALA98, ARG222, PRO223, VAL224, PHE281, LEU499
Licochalcone a	− 8.6	ALA99, THR310	ALA98, PRO223, VAL224, ILE282, VAL285, TYR314, ILE445, PHE446, PHE450
Rivularin	− 8.5	ARG449, ASP533	ALA98, ILE282
Acacetin	− 8.3	ALA99, ARG222, GLN495, SER497	ALA98, VAL224, TYR503
Baicalein	− 8.3	TYR314	ILE282, TYR503, MET532

**Table 4:** The docking results of top-ranked 5 compounds towards RORA

Compound	Docking score (kcal/mol)	H-Bond interactions	Hydrophobic interactions
Stigmasterol	− 11.6	GLN289	TRP320, CYS323, ILE327, VAL364, PHE365, MET368, VAL379, PHE391, LEU394, CYS396, PHE399, VAL403, HIS484, LYS487
beta-sitosterol	− 11.2	GLN289	TRP320, CYS323, ILE327, ALA330, VAL364, PHE365, MET368, VAL379, PHE391, LEU394, CYS396, PHE399, VAL403, HIS484, LYS487
sitosterol	− 11.2	GLN289	TRP320, CYS323, ILE327, ALA330, VAL364, PHE365, MET368, VAL379, PHE391, LEU394, CYS396, PHE399, VAL403, HIS484, LYS487
Euchrenone	− 10.6	ASP382	CYS323, ILE327, ALA330, VAL364, PHE365, ARG367, ALA371, PHE399, ILE400, VAL403
Kanzonol B	− 9.5	GLN289, LYS326	LYS326, ILE327, PHE365, PHE391, PHE399, ILE400, VAL403

**Table 5:** The docking results of top-ranked 5 compounds towards REV-ERBα

Compound	Docking score (kcal/mol)	H-Bond interactions	Hydrophobic interactions
Euchrenone	− 8.1	LYS605	VAL447, VAL451, LYS473, LEU607, PHE609
Daidzein-4,7-diglucoside	− 8.0	ARG461, GLN465, GLN468, LYS473, GLU604	VAL451, VAL469, LEU472, LYS473
Kanzonol B	− 7.5	SER603	VAL447, LEU606, LEU607, PHE609
glyasperin F	− 7.3	LYS605	VAL447, LEU607
oroxylin a	− 7.1	LEU472, PHE477	VAL447, LEU607

### Molecular dynamic simulation study revealed the mapping ligand binding sites and affinity for complexes

**Fig. 6:**
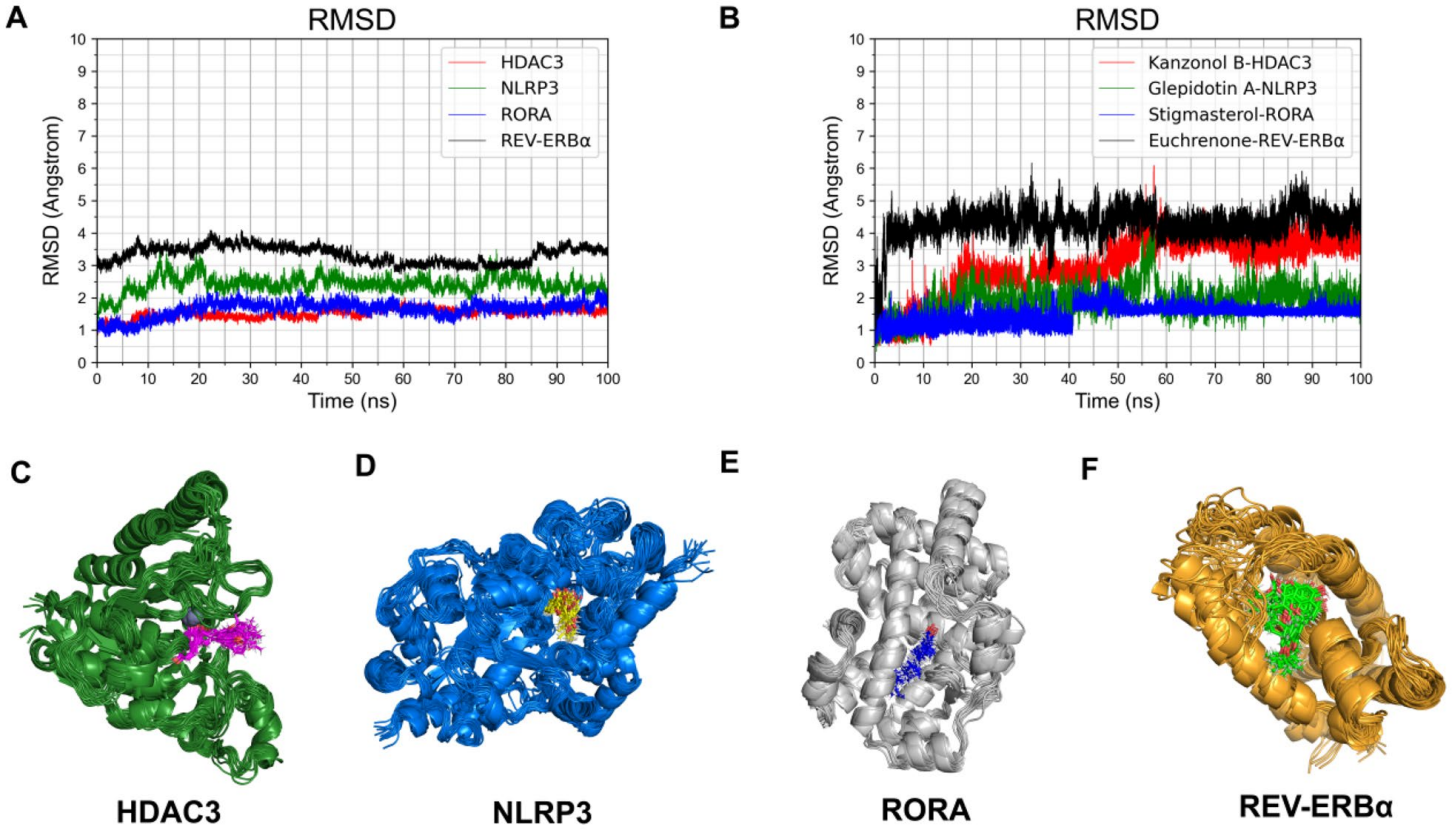
The molecular dynamics simulation results of selected compounds in GGQLD with HDAC3, NLRP3, RORA and REV-ERBα. **A** RMSD values of four protein backbones through 100 ns MD simulation. **B** RMSD values of ligands through 100 ns MD simulation. **C** Snapshot of docking poses of Kanzonol B with HDAC3 during 100 ns MD simulation. **D** Snapshot of docking poses of Glepidotin A with NLRP3 during 100 ns MD simulation. **E** Snapshot of docking poses of Stigmasterol with RORA during 100 ns MD simulation. **F** Snapshot of docking poses of Euchrenone with REV-ERBα during 100 ns MD simulation. Red, green, blue and black lines represent HDAC3/Kanzonol B, NLRP3/Glepidotin A, RORA /Stigmasterol and REV-ERBα/Euchrenone complexes, respectively. Representations, coloring and display of ligands and residues are as figures

In order to assess the stability of the four selected ligands with their corresponding targets, we conducted 100 ns MD simulations on the HDAC3/Kanzonol B, NLRP3/Glepidotin A, RORA/Stigmasterol, and REV-ERBα/Euchrenone complexes, and explored RMSD values. Figure 6 presents the detailed results. RMSD values for HDAC3, NLRP3, and RORA protein backbones were mostly below 3 Å, indicating high stability of their structures during the simulation, except for REV-ERBα (Fig. 6A). This suggests that the conformation of REV-ERBα protein undergoes significant changes when bound to ligands, as its initial crystal structure used for molecular docking did not bind to the ligand but to NCOR1 protein, as previously proven by Phelan, C.A [26]. In this study, a semi-flexible docking approach was used to perform molecular docking. During the process, the protein structure was kept rigid, which resulted in a significant change in RMSD values for REV-ERBα in the initial stage of the simulation. This indicates that the structure of REV-ERBα changed to adapt to the new ligand molecule, which makes the relatively high RMSD values reasonable. For the HDAC3/Kanzonol B complex, the RMSD values of Kanzonol B increased to around 3 Å in the first 20 ns, remained relatively stable until 50 ns, and fluctuated before finally stabilizing at around 4 Å (Fig. 6B). The stable interaction of Kanzonol B with Zn^2+^ ions in the HDAC3 pocket was observed in Fig. 6C, with the Cap group of Kanzonol B swinging due to its flexible structure. Similarly, Glepidotin A and Stigmasterol were observed to stably bind to the active sites of NLRP3 and RORA, respectively (Fig. 6D and E). The RMSD values of Glepidotin A in NLRP3 remained stable at around 2 Å in the last 80 ns, except for a slight increase in the first 20 ns (Fig. 6B). The RMSD values of Stigmasterol in RORA remained stable at about 1.5 Å in the first 40 ns and around 2 Å in the next 60 ns. For the REV-ERBα/Euchrenone complex, the RMSD values of the ligand suddenly increased to 4 Å in the first 2 ns and remained relatively stable thereafter. Combining the RMSD values of the four ligands, it can be found that the RMSD value of Euchrenone is relatively large. However, as mentioned above, when the ligand binds to REV-ERBα, the structure of the protein changes appropriately, and the conformation of the ligand also changes. Therefore, it is relatively reasonable for Euchrenone to have relatively large RMSD values. In Fig. 6F, it can be seen that the conformation of the Euchrenone at 0 ns is different from that at other times, and the conformations at other times basically overlap, which is consistent with the RMSD data. Therefore, the stable binding of the four selected ligands to the corresponding proteins can be reasonably concluded based on the observed results.

## Discussion

The therapeutic effects of GGQLD in reducing SUA levels and improving the metabolic activity of HUA patients were observed. To investigate the underlying mechanism, network pharmacology and transcriptomic analyses of PTECs stimulated with GGQLD were conducted, revealing its potential to modulate the expression of circadian clock genes, including REV-ERBα, HDAC3, NFIL3, CYR2, and PER2, and the expression of RORA involved in ILCs development for HUA treatment. Further comparison of ILC subsets and the relative expression of circadian clock genes in PBMCs of asymptomatic HUA patients before and after GGQLD treatment indicated that REV-ERBα exhibited the strongest correlation with SUA levels. Moreover, GGQLD was found to regulate the number of ILCs in the PBMCs of asymptomatic HUA patients.

HUA may trigger the formation of monosodium urate crystals which can act as danger signals to the immune system. This can activate the NLRP3 inflammasome leading to the production of IL-1β and IL-18 in response to external stimuli. These factors play a role in mediating inflammation, pyroptotic cell death, and necroinflammation  [15]. Research has shown that elevated SUA levels can activate the TLR4/NLRP3 pathway, leading to epithelial inflammation. Our previous study also demonstrated that increased soluble urate levels can activate the NLRP3 inflammasome and increase the IL-1β gene expression ratio [27]. In this study, ELISA analysis revealed elevated levels of pro-inflammatory cytokines IL-18 and IL-1β in the plasma of asymptomatic HUA patients, which were positively correlated with SUA content. Notably, after GGQLD treatment, the levels of IL-18 and IL-1β in HUA patients were significantly decreased. Our previous studies have also shown that GGQLD treatment can reduce the expression of NLRP3. Using molecular docking technology, we further investigated the interaction between 40 compounds in GGQLD and NLRP3, providing additional evidence that GGQLD can alleviate the inflammatory microenvironment in HUA patients.

Recent studies have demonstrated that IL-1β and IL-18 can induce the secretion of pro-inflammatory factors by innate lymphoid cells, leading to the development of a microinflammatory environment [28, 29]. A recent study has indicated a correlation between HUA and innate immune cells [13]. Following brief stimulation, innate immune cells can acquire a persistent hyperresponsive phenotype [30]. Monocytes and macrophages can develop a memory response to both exogenous pathogens and endogenous molecules such as UA [31]. In male patients with asymptomatic HUA, there is a correlation between the level of SUA and the count of NKG2D + NK cells, as observed by some researchers [15]. We observed an increased proportion of ILCs in PBMCs, as well as an increased ILC3-ILC2 ratio in asymptomatic HUA patients, which could be attributed to the overexpression of pro-inflammatory cytokines, such as IL-1β and IL-18. These cytokines can activate the abnormal secretion of cytokines by innate lymphocytes, driving continuous inflammatory responses [30].

GGQLD, a traditional Chinese medicine prescription, is widely used to treat inflammatory disorders. GGQLD has been shown to alleviate ulcerative colitis by inhibiting the IL-6/JAK2/STAT3 signal transduction pathway and restoring the homeostasis of Th17 and Treg cells within colon tissues [32]. Additionally, it has been suggested that GGQLD could potentially alleviate non-alcoholic fatty liver disease (NAFLD) and hepatic steatosis by regulating inflammatory factors and oxidative stress through modulation of the NLRP3 signal axis [33]. Our results revealed a decrease in the number of ILCs and ILC3-ILC2 ratio after GGQLD treatment, which could be attributed to the reduction of IL-1β and IL-18 levels. This suggests that GGQLD might have a potential role in inhibiting the production of pro-inflammatory factors and regulating ILCs function to alleviate the inflammatory state in asymptomatic HUA patients.

Research studies have shown that ILC2s subsets can protect against kidney injury [34, 35], and the levels of ILC2s were inversely associated with the prognostic biomarkers of chronic kidney disease [36]. Research has indicated that the IL-33-ILC2 pathway has a crucial protective role in renal ischemia–reperfusion injury (IRI) and may be considered a potential therapeutic intervention  [37]. Our study revealed that middle-aged and elderly asymptomatic HUA patients had a higher ILC3-ILC2 ratio. However, after treatment with GGQLD, the proportion of ILC3 in PBMCs and ILC3-ILC2 ratio decreased, suggesting that GGQLD not only alleviates inflammation but also regulates the differentiation and transformation of ILCs, thereby protecting against HUA-induced kidney injury. Our study is the first to report that GGQLD modulates ILCs function for immune protection. Furthermore, we found that many components of GGQLD interact with RORA, a critical gene responsible for ILC development and maturation. Stigmasterol, an active component of GGQLD, has been shown to reduce neuroinflammation and regulate the immune response [38, 39]. Molecular docking analysis revealed that Stigmasterol binds stably to the active site of RORA, which is an important transcription factor involved in the development of ILC2s.

Mutations in circadian genes can impact immune cell function and play a significant role in the development and advancement of various metabolic disorders [40]. Currently, there is limited research on the association between HUA and circadian rhythm. A recent study found that a longer daytime nap (rather than nighttime sleep) was independently linked to an increased risk of HUA among the Chinese population [41]. In this study, we discovered for the first time that GGQLD may be effective in treating HUA by regulating clock genes such as REV-ERBα, HDAC3, NFIL3, CYR2, and PER2 through RNA-seq analysis in PTECs. We confirmed significant differences in clock genes between HUA patients and non-HUA patients in PBMCS through RT-qPCR analysis. Cluster analysis was used to explore the relative expression of circadian clock genes in PBMCs of NC, HUA before GGQLD treatment, and HUA after GGQLD treatment patients. Our results indicated that the HUA after GGQLD treatment group and NC group were clustered into one group, confirming that GGQLD might regulate circadian clock genes in asymptomatic HUA patients. Furthermore, previous research by Qiu Z et al. demonstrated that HDAC3 is an important component of the circadian negative feedback loop, controlling the expression of REV-ERBα, a circadian nuclear receptor, to maintain circadian gene stability, such as BMAL1 [42]. According to Wu and colleagues, it has been demonstrated that a disturbed circadian clock is correlated with exhaustion of T cells [43]. The present study observed a significant decrease in the relative RNA expression of REV-ERBα in peripheral blood mononuclear cells (PBMCs) obtained from patients with hyperuricemia (HUA), and this decrease was correlated with the level of serum uric acid (SUA). However, following treatment with GGQLD, there was a noteworthy increase in the RNA expression of REV-ERBα. Furthermore, our study revealed that Kanzonol B, a compound known for its anti-neuroinflammatory properties due to its ability to inhibit the production of NO and PGE2 [44], exhibited a high binding affinity for HDAC3, as evidenced by its relatively high docking score in our analysis.

REV-ERBα, a crucial component of the circadian clock gene, serves as a transcriptional suppressor and plays a significant role in regulating the molecular clock [45]. REV-ERBα, being an integrator of the circadian clock and cell metabolism, is known to have a direct influence on metabolic genes [46]. Owing to its critical role in metabolic syndromes and sleep disorders, it has been identified as a promising target for drug development [47]. Several studies have demonstrated that REV-ERBα can ameliorate various diseases by modulating the inflammatory pathway and regulating immune cells  [48, 49]. ILC3 cells exhibit a circadian rhythm and any disturbance in this rhythm can result in cytokine imbalance [50], thereby promoting inflammatory responses [51]. Our study has demonstrated that the peripheral blood ILC3-ILC2 ratio is elevated in patients with hyperuricemia (HUA), and there is a significant alteration in the expression of the circadian clock gene, particularly REV-ERBα. These findings led us to speculate that the dysregulation of the circadian clock gene in HUA patients could disrupt the function of ILCs and lead to further exacerbation of inflammation. Moreover, REV-ERBα has been shown to bind to heme in a reversible manner, thereby coordinating circadian rhythm, glucose and lipid metabolism, and energy homeostasis [26. Our molecular docking results provide evidence that several compounds present in GGQLD can interact with REV-ERBα and contribute to the coordinated regulation of circadian rhythm. Specifically, Euchrenone has been shown to bind to the ligand binding domain of REV-ERBα, resulting in conformational changes and promoting the recruitment of transcriptional co-regulatory proteins to receptor-specific gene promoter complexes. This, in turn, inhibits the transcription of NLRP3.

However, our study has some limitations. Firstly, we did not collect blood samples from all patients at different times, which could affect the interpretation of the effect of the circadian clock gene on HUA patients. Secondly, the study population consisted majority of middle-aged and elderly men, which may not be representative of the general population. Lastly, due to the reluctance of patients to participate in the blood withdrawal required for biochemical indicators testing, the sample size was relatively small.

In summary, our study has shown that GGQLD can effectively treat asymptomatic hyperuricemia (HUA) patients by modulating the expression of circadian clock genes and regulating the proportion of ILC cells, as supported by our clinical data and in vitro experiments. Moreover, our molecular docking and dynamics simulations have provided further evidence of the compounds' interactions with inflammatory bodies and molecular proteins involved in regulating circadian rhythm and innate immunity. These findings not only provide valuable insights into the therapeutic targets for HUA but also establish a theoretical foundation for its treatment. Future studies with larger sample sizes and wider age ranges will help elucidate the underlying molecular mechanisms.

## Supplementary Information

**Additional file 1: Table S1.** Clinical characteristics of the study participants. **Figure S2.** HPLC analysis of stigmasterol. **Figure S3.** Flowchart process of ILC. **Table S4.** Primers for RT-PCR.

## Data Availability

Data analyzed in this study can be obtained from the corresponding author upon request.

## Abbreviations

GGQLD: Gegen Qinlian Decoction
HUA: Hyperuricemia
PBMCs: Peripheral blood mononuclear cells
PTECs: Proximal tubule epithelial cells
HPLC: High-Performance Liquid Chromatography
DEGs: Differentially expressed genes
MD: Molecular dynamics
ILCs: Innate lymphoid cells
HDAC3: Histone Deacetylase 3
NLRP3: NOD-like receptor protein 3
RORA: RAR-related orphan receptor A
REV-ERBα: Nuclear receptor subfamily 1
ILCP: ILC progenitor
UA: Uric acid
NK: Natural killer
HPLC: High-Performance Liquid Chromatography
SUA: Serum uric acid
CCK8: Cell Counting Kit-8
FDR: False discovery rate
FC: Fold change
PPI: Protein–protein interaction
EDTA: Ethylenediamine tetraacetic acid
FA: Fluorescent antibody
ELISA: Enzyme-linked Immuno Sorbent Assay
CGenFF: CHARMM General Force Field
PME: Particle mesh Ewald
RMSD: Root-mean-square deviation
IBD: Inflammatory bowel disease
TB: Tuberculosis
GVHD: Graft-versus-host disease
