# Supplementary material for: Gegen Qinlian Decoction treatment of asymptomatic hyperuricemia by targeting circadian immune function
Source: Chin Med. 2023 Jun 27;18:77. doi: 10.1186/s13020-023-00775-z (PMC10304353; doi:10.1186/s13020-023-00775-z)
Supplement: Supplementary file 1 — Additional file 1: Table S1. Clinical characteristics of the study participants. Figure S2. HPLC analysis of stigmasterol. Figure S3. Flowchart process of ILC. Table S4. Primers for RT-PCR. [file 13020_2023_775_MOESM1_ESM.docx]

**Supplement 1. Clinical characteristics of the study participants**

|  | Normal control  (N=25) | | HUA before GGQLD  (N=32) | HUA after GGQLD  (N=32) | *P*-value |
| --- | --- | --- | --- | --- | --- |
| Age | 70.880±6.037 | 67.250±8.332 | | | 0.1461 |
| Gender (Male/Female) | 21/4 | 27/5 | | | 0.0057* |
| BMI (kg/m2) | 24.229±4.639 | 22.473±4.610 | | | 0.2844 |
| SUA (umol/L) | 286.320±52.760 | 496.100±39.430 | | 417.900±69.620 | 0.0085* |
| UUA (mmol/24h) | 3.976±1.074 | 3.527±1.183 | | 3.934±1.468 | 0.3169 |
| SCR (umol/L) | 68.150±12.540 | 85.650±14.070 | | 83.150±12.530 | <0.0001* |
| eGFR (ml/min) | 102.900±14.84 | 86.99±16.51 | | 88.55±14.46 | 0.0003* |
| TG (mmol/L) | 1.163±0.611 | 1.911±0.981 | | 1.771±0.706 | 0.0018* |
| TC (mmol/L) | 4.592±0.701 | 4.864±1.149 | | 4.520±0.9300 | 0.3307 |
| ALT (U/L) | 21.810±7.806 | 31.550±27.260 | | 24.45±14.550 | 0.1324 |
| AST (U/L) | 19.160±4.660 | 24.640±12.720 | | 21.560±7.605 | 0.0858 |

Abbreviations: BMI, body mass index; SUA, serum uric acid; UUA, urine uric acid; Scr, serum creatinine; eGFR, estimated glomerular filtration rate; TG, triglyceride; TC, total cholesterol; ALT, alanine aminotransferase; AST, aspartic acid transferase. *, *P*≤0.05

**Supplement 2. HPLC analysis of stigmasterol**

**
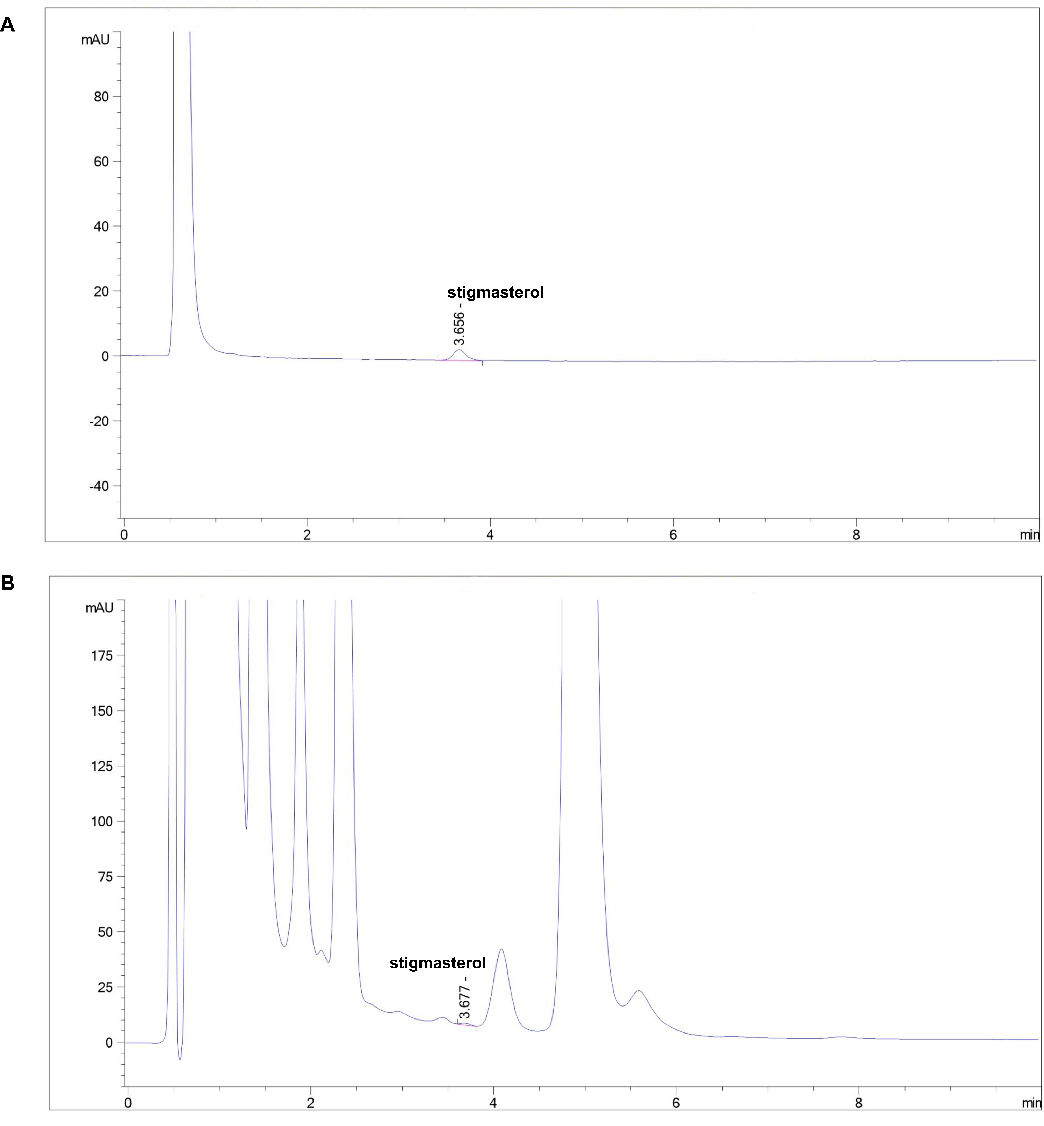
**

(A) Analysis of standard products; (B) Analysis of the sample.

**Supplement 3. Flowchart process of ILC**

**
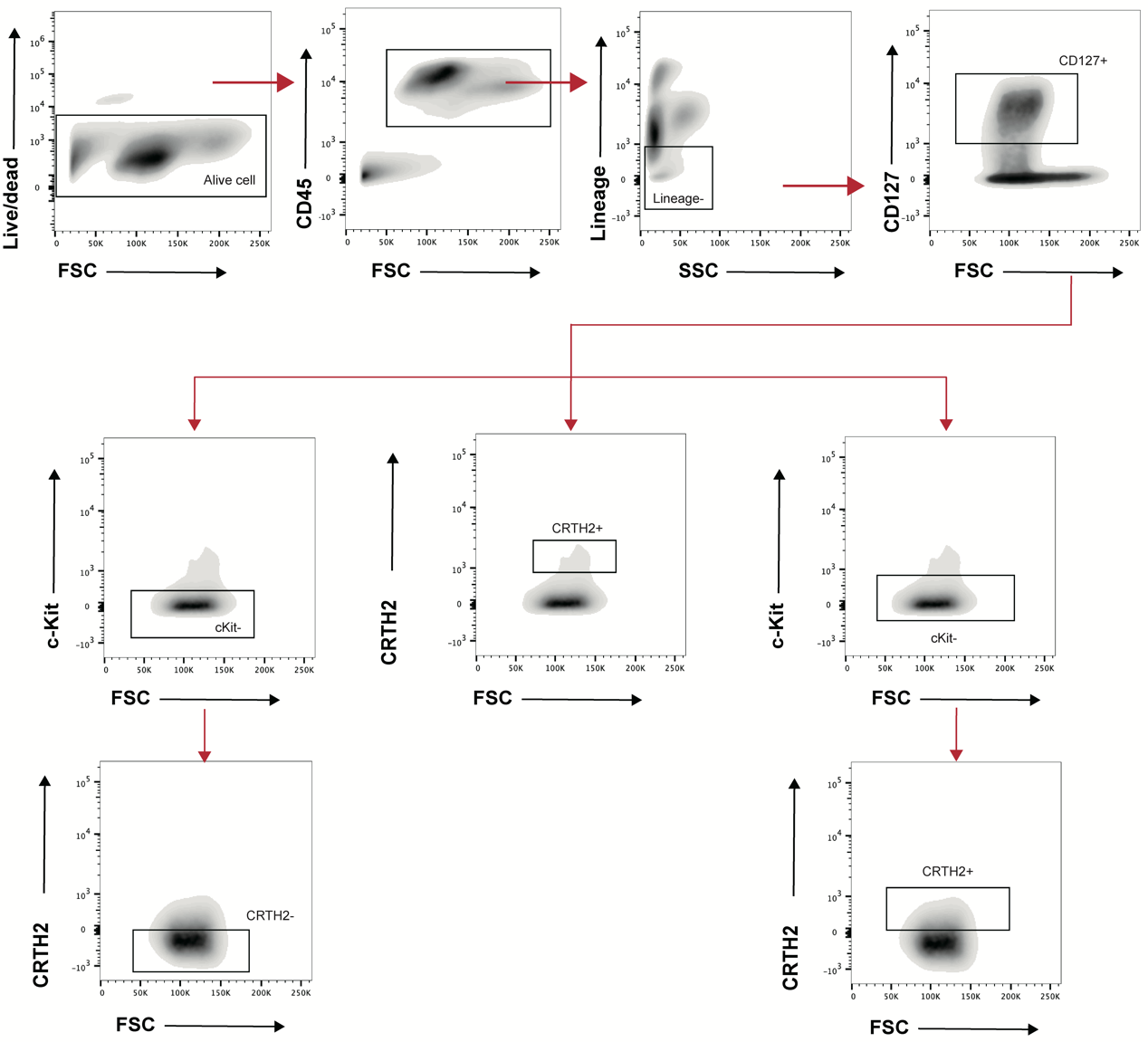
**

**Supplement 4. Primers for RT-PCR**

| Gene | Forward Primer | Reverse Primer |
| --- | --- | --- |
| β-actin | GTGGACATCCGCAAAGAC | AAAGGGTGTAACGCAACTA |
| NFILC3  HDAC3  REV-ERBα  PER2  CLOCK  CRY2 | GCTACACAAGGGAGCCAAGAGATG  AGACATCGCTGCTGGTAGAAGAGG  ATCTTCCTCGTCGTCATCCTCCTC  ACCTGACCTCGCTGGCACTG  GTGACTGCTCCTGTAGCTTGTGG  CTCTCCTGCCGCCTCTTCTACTAC | GAGTTGCTGGAGGATCGGTTGAC  GGGTGCTGACATCTGGATGAAGTG  TGGTGATGTTGCTGGTGCTCTTG  CGGCTGCGGCTTCTTGTCTC  TGCTGCTGCTGCTGCGTTAC  GGTTGTTGGTAGCTGCCGTGTAG |
